# Characterisation of Brachycephalic Obstructive Airway Syndrome in French Bulldogs Using Whole-Body Barometric Plethysmography

**DOI:** 10.1371/journal.pone.0130741

**Published:** 2015-06-16

**Authors:** Nai-Chieh Liu, David R. Sargan, Vicki J. Adams, Jane F. Ladlow

**Affiliations:** 1 Department of Veterinary Medicine, University of Cambridge, Cambridge, Cambridgeshire, United Kingdom; 2 Vet Epi Limited, Birmingham, West Midlands, United Kingdom; University of Missouri, UNITED STATES

## Abstract

Brachycephalic obstructive airway syndrome (BOAS) is an important health and welfare problem in several popular dog breeds. Whole-body barometric plethysmography (WBBP) is a non-invasive method that allows safe and repeated quantitative measurements of respiratory cycles on unsedated dogs. Here respiratory flow traces in French bulldogs from the pet population were characterised using WBBP, and a computational application was developed to recognise affected animals. Eighty-nine French bulldogs and twenty non-brachycephalic controls underwent WBBP testing. A respiratory functional grading system was used on each dog based on respiratory signs (i.e. respiratory noise, effort, etc.) before and after exercise. For development of an objective BOAS classifier, functional Grades 0 and I were considered to have insignificant clinical signs (termed here BOAS-) and Grades II and III to have significant signs (termed here BOAS+). A comparison between owner-perception of BOAS and functional grading revealed that 60 % of owners failed to recognise BOAS in dogs that graded BOAS+ in this study.WBBP flow traces were found to be significantly different between non-brachycephalic controls and Grade 0 French bulldogs; BOAS- and BOAS+ French bulldogs. A classifier was developed using quadratic discriminant analysis of the respiratory parameters to distinguish BOAS- and BOAS + French bulldogs, and a BOAS Index was calculated for each dog. A cut-off value of the BOAS Index was selected based on a receiver operating characteristic (ROC) curve. Sensitivity, specificity, positive predictive value, and negative predictive value of the classifier on the training group (n=69) were 0.97, 0.93, 0.95, and 0.97, respectively. The classifier was validated using a test group of French bulldogs (n=20) with an accuracy of 0.95. WBBP offers objective screening for the diagnosis of BOAS in French Bulldogs. The technique may be applied to other brachycephalic breeds affected by BOAS, and possibly to other respiratory disease in dogs.

## Introduction

Brachycephalic Obstructive Airway Syndrome (BOAS) is a common respiratory disorder in brachycephalic (i.e. short-skulled, flat-faced) canine breeds such as pugs, French bulldogs (FB), and bulldogs. Excessive breeding selection for brachycephaly has led to deformation in the upper respiratory tract and subsequent airway obstructions because the soft tissues are not reduced in the same proportion as the skull [1,2]. Primary lesions may include an oversized soft palate, stenotic nares, redundant pharyngeal folds, deviated nasal septum, aberrant conchal growth, hypertrophic tonsils, hypoplastic trachea, and macroglossia. Secondary lesions include everted laryngeal saccules and laryngeal collapse. BOAS-affected dogs may also display a variety of clinical signs such as noisy and laboured breathing, regurgitation/vomiting, heat and exercise intolerance, cyanosis, and collapse. The clinical signs are usually chronic and deteriorate with time if the lesions are left untreated [1–6].

Welfare concerns about brachycephalic breeds have been raised recently due to the soaring popularity and the assumed high prevalence of BOAS. The FB, for example, has within the past-ten years gone from the 76^th^ (324 FB registered, 2005) to the 4^th^ (9670 FB registered, 2014) most popular breed registered in the UK with, in addition, a large number of unregistered dogs being imported [7]. The severity of the respiratory compromise associated with BOAS is reported to be increasing [5]. However the syndrome lacks a single distinguishing feature and is usually identified by the presence of a combination of clinical signs and laboratory manifestations. The only well described clinical grading system for BOAS is based on history as reported by dog owners in terms of the type and frequency of respiratory signs [4]. Unfortunately, low disease recognition by owners makes it likely that BOAS in brachycephalic dogs is significantly under-diagnosed [8]. A lack of objective data on respiratory function makes it difficult to monitor the presence or progress of the disease. To minimise the welfare impact on the increased population of affected brachycephalic dogs, and to inform therapeutic decisions, further characterisation of respiratory parameters in the disease and a specific screening test for BOAS are required.

Non-invasive respiratory measurements previously used in dogs include a face mask and pneumotachograph, used to develop tidal breathing flow volume loops (TBFVL) for assessment of airway obstruction [9–12]. However, practical issues make it difficult to establish a ‘gold standard’ diagnostic test for BOAS in client-owned dogs. Designing a face mask that forms an airtight seal on brachycephalic dogs is technically difficult, leading to increased dead space and leakage that may cause underestimation of flow rates. Having a mask over the face may also result in stress in brachycephalic dogs, changing the respiratory waveforms [9]. Head-out whole body plethysmography (HOP) and spirometry encounter the same problem as the use of a face mask is required [13–16].

Whole-body barometric plethysmography (WBBP) is a non-invasive and objective technique to measure respiratory function while the animal is fully conscious and minimally restrained [17–23]. The animal rests in the WBBP chamber and respiration causes barometric pressure oscillations proportional to tidal volume. Pumped bias airflow maintains carbon dioxide levels, temperature and humidity at normal ranges enabling long-term measurements [24]. In previous studies using WBBP to assess respiratory function in 11 brachycephalic dogs [17] and other canine breeds [19,21,22,25], sedation has typically been used to reduce panting and movement making WBBP easier to interpret. However, a significant effect of sedation on respiratory parameters in experimental beagles was reported and the muscle relaxant properties of sedation could exacerbate any existing upper airway obstruction [19,26–28].

A modified WBBP protocol using unsedated dogs has been tested by the authors on over 620 untrained dogs among 41 breeds. Stress behaviours were not observed in the large majority (see also Hirt et al., 2007) [29]. This non-invasive method can be used to screen brachycephalic canine populations as a routine respiratory function assessment. The method was therefore adopted for the present study.

The aims of the study were to (1) characterize respiratory cycles using WBBP in non-brachycephalic control dogs and FB with a range of respiratory compromise; (2) construct and validate a model and a score system (BOAS Index) to discriminate the respiratory cycles of FB with moderate/severe BOAS from those without significant clinical signs. It is proposed that a WBBP test followed by computational analysis of respiratory cycles would be a useful tool in identifying FB with moderate/severe BOAS that are not suitable for breeding and require medical attention.

## Materials and Methods

### Subjects

‘Patient FB’ (i.e. FB that were referred for BOAS consultation at the Queen’s Veterinary School Hospital, QVSH) and ‘study FB’ (i.e. FB volunteered by UK owners and breeders) were recruited to the study between September 2011 and November 2014. Non-brachycephalic dogs referred to QVSH for other than respiratory diseases, and non-brachycephalic staff owned dogs, were included in this study as controls. The Department of Veterinary Medicine, University of Cambridge approved this study under informed ethical consents, CR62 and CR63. All dog owners gave informed consent for the inclusion of their animals in the study. A detailed history of the dog was taken from the owners, including type, severity, and frequency of respiratory signs, while awake at rest, while sleep, and during physical exercise (S1 File). Body weight (BW), body condition score (BCS, a standard assessment of whether animals are underweight or overweight by use of a nine-point scale), gender and age were noted. Dogs that had been diagnosed with overt lower airway disease and/or were younger than one year old were excluded from the study.

### Functional Grading System for BOAS

A functional grading system of BOAS severity was designed for an initial comparison of respiratory performance amongst dogs, and for further use in training the computational classifier on WBBP data (Table 1). The functional grading system was based on clinical evaluation before and after a 3-minute exercise tolerance test (ETT) with trotting speed of approximately 4–5 miles per hour performed by the study investigators. Each dog was assigned with a BOAS function grade: Grade III as severe BOAS, where the owners are advised that the dogs require immediate surgical intervention; Grade II as moderate BOAS, in which the dogs have clinically relevant disease, requiring medical attention (i.e. management and/or surgical intervention); Grade I as mild BOAS, in which the dogs show mild stertorous noise but exercise tolerance is unaffected. Grade 0 dogs show no signs of BOAS and are considered BOAS free.

**Table 1:** Functional grading system of brachycephalic obstructive airway syndrome (BOAS) based on respiratory signs before and after an exercise tolerance test (ETT).

		Respiratory noise ^a^	Inspiratory effort^b^	Dyspnoea/ Cyanosis/ Syncope^c^
**Grade 0**	**Pre-ETT**	Not audible	Not present	Not present
	**Post-ETT**	Not audible	Not present	Not present
**Grade I**	**Pre-ETT**	Not audible or mild	Not present	Not present
	**Post-ETT**	Mild	Not present to mild	Not present
**Grade II**	**Pre-ETT**	Mild to moderate	Mild to moderate	Not present
	**Post-ETT**	Moderate to severe	Moderate to severe	Mild dyspnoea; cyanosis or syncope not present
**Grade III**	**Pre-ETT**	Moderate to severe	Moderate to severe	Moderate to severe dyspnoea; may or may not present cyanosis. Inability to exercise.
	**Post-ETT**	Severe	Severe	Severe dyspnoea; may or may not present cyanosis or syncope.

The clinical grading was based on respiratory signs before (pre-ETT) and immediately after the exercise tolerance test (post-ETT) as described in the methods section.

^a^ Respiratory noise was diagnosed by pharyngolaryngeal auscultation. Mild: only audible under auscultation; moderate: intermittent audible noise that can be heard without stethoscope; severe: constant audible noise that can be heard without stethoscope.

^b^ An abnormal respiratory cycle characterized by evidence of increased effort to inhale the air in with the use of diaphragm and/or accessory muscles of respiration and/or nasal flaring with an increase in breathing rate. Mild: regular breathing patterns with minimal use of diaphragm; moderate: evidence of use of diaphragm and accessary muscles of respiration; severe: marked movement of diaphragm and accessary muscles of respiration.

^c^ Dogs that have had episodes of syncope and /or cyanosis as documented by owner’s report are classified into Grade III without ETT. Mild dyspnoea: presents sign of discomfort; Moderate dyspnoea: irregular breathing, signs of discomfort; severe dyspnoea: irregular breathing with signs of breathing discomfort and difficulty in breathing.

### Data Collection: Non-invasive Respiratory Function Test Using WBBP

**Fig 1:**
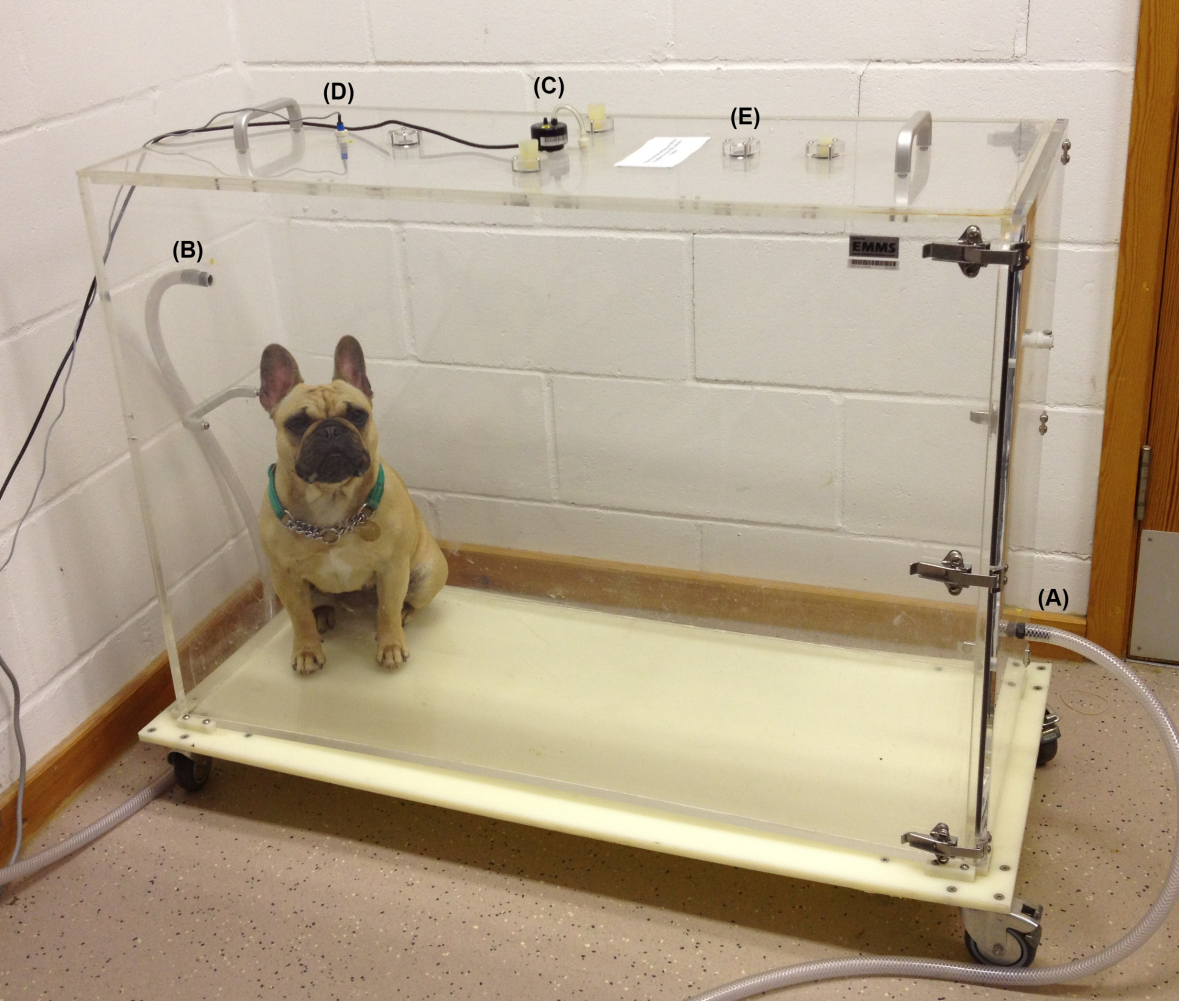
The chamber used for whole-body barometric plethysmography (WBBP) with a French bulldog undergoing the test. A pole of the chamber pressure differential transducer is opened to the top of the chamber (C); two inlets (A and B) are connected to the front and back of the chamber in order to ventilate with a bias flow of room air (20 L/minute); an audio sensor is located on the top of the chamber (D); together with pneumotachograph screens (E).

WBBP was performed by placing the dog into a transparent Perspex chamber (Model PLY-360 for dogs, EMMS) with balanced airflow (20L/min) and an inner volume of 280.0 L (100 cm x 40 cm x 70 cm) (Fig 1). A pressure differential transducer (TPF-100, EMMS) was attached to the top of the chamber. Transduced signals were amplified using a strain gauge amplifier and digitized using commercial software (ESS-102 EMMS Data Acquisition, eDacq, for Microsoft Windows XP). Dynamic calibration of the box pressure signal was performed before each test by injecting 50 mL of room air via a syringe into the chamber and integrating under the resultant flow curve. The chamber used the exterior room air as a reference. After the dog was acclimatised to the testing environment for 5–10 minutes, measurements were recorded for 20 minutes. Subject behaviour was monitored using a digital camera. Any dog that did not tolerate the test (i.e. showed signs of anxiety) for 5 minutes during the first recording was removed, and given a second attempt when calm. Dogs that were not tolerant after two acclimatising sessions were excluded from this study.

**Fig 2:**
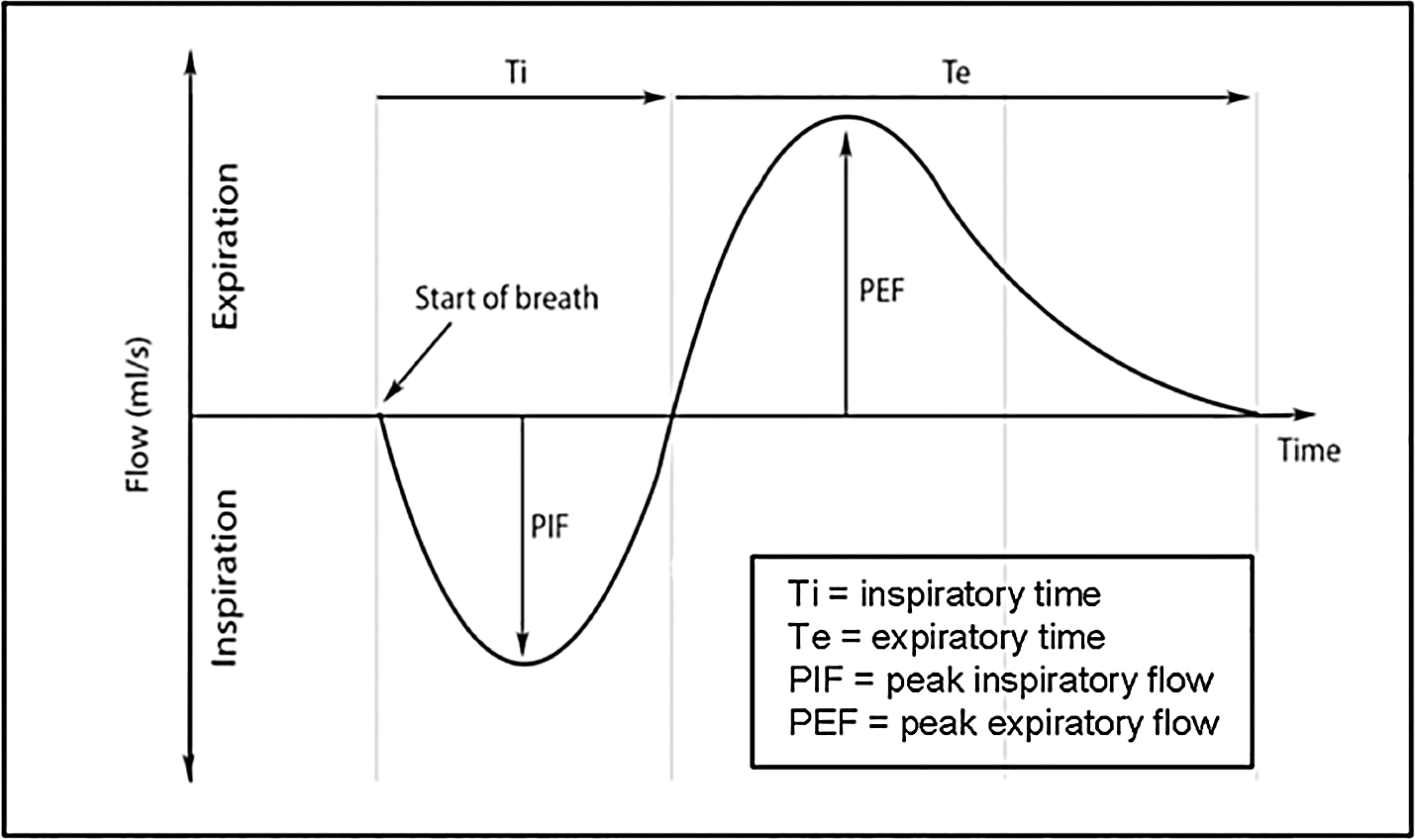
WBBP flow waveform illustration for a single respiratory cycle. **The** flow cycle starts from inspiration (below the zero line of flow rate) then expiration (above the zero line of the flow rate).

An illustration of a WBBP flow waveform for a single breath cycle is shown in Fig 2. Selected respiratory parameters: tidal volume (TV), inspiratory time (Ti), expiratory time (Te), peak inspiratory flow rate (PIF), peak expiratory flow rate (PEF), respiratory rate (RR), and minute ventilation (MV) were used to characterise the WBBP flow waveform. The data processing procedures involved in quantifying a plethysmographic flow waveform comprised: automatically marking the start and finish of each breath cycle using eDacq software; the extraction of trace features associated with body movement or vocalization according to the video recording; and manual exclusion of incorrectly computer-detected respiratory cycles. Breath cycles used in the study were those in which the difference between inspiratory volume and expiratory volume were balanced within 20% and the dog was relaxed and still, and was not panting. The first 20 such breath cycles during wakefulness in each dog’s record were used for subsequent analysis.

### Statistical Analysis, Classifier Design, and the Derivation of a BOAS Index

The respiratory functional consequences of a Grade I assessment for the dog are very minor, so for binary comparisons between functionally BOAS—and BOAS + FB, ‘Grade 0 and Grade I FB’ were compared with ‘Grade II and Grade III FB’. In the remainder of the article Grade0/I will be referred to as BOAS- and Grade II/III as BOAS+. BOAS- FB were compared with BOAS+ FB on gender using Pearson’s chi-square tests; on age and BCS using independent t-test. Statistical tests were conducted using SPSS (IBM, version 22.0 for Mac). A p value <0.05 was considered statistically significant.

After a pilot investigation PEF/PIF, Te/Ti, and MV/BW were used in the proposed classifier. These parameters were previously reported as indicators of upper airway functionality using TBFVL and also showed significant differences in the pilot study [9,10]. Initial overview of the distribution of these parameters suggested that respiratory cycles of functional BOAS+ FB show higher variation between dogs and within dog than did those of BOAS- FB. Therefore the means and standard deviations of each of the parameters were derived from 20 representative breaths selected for each dog. Normality of the data within groups was tested by Kolmogorov-Smironov (K-S) test; Homogeneity of variance was tested by Lavene’s test. Independent t-tests were used to compare the means between groups: (1) non-brachycephalic controls versus Grade 0 FB controls; (2) BOAS- FB versus BOAS+ FB.

**Fig 3:**
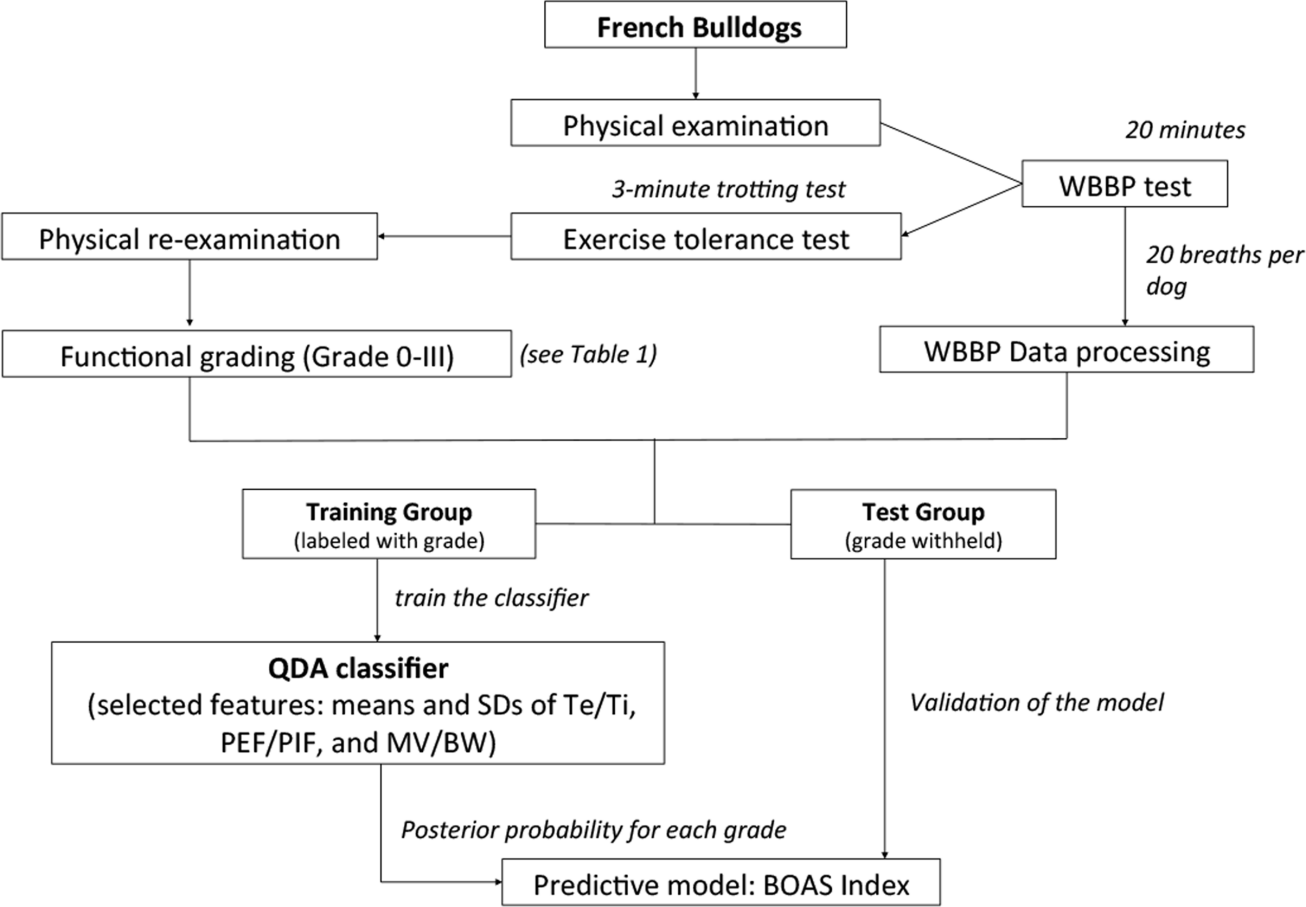
A flowchart of the study design. WBBP = whole body barometric plethysmography; BOAS = brachycephalic obstructive airway syndrome; QDA = quadratic discriminant analysis; SD = standard deviation; Te/Ti = expiratory time/ inspiratory time; PEF/PIF = ratio of peak expiratory flow to peak inspiratory flow; MV/BW = minute volume / body weight.

A summarised flow-chart for classifier design is shown in Fig 3. The FB subjects were divided into 2 groups: a training group and a test group of 20 more recently recruited dogs. The dogs in the training group were tagged with the appropriate labels (i.e. the functional grading results) and used to develop and train the classification algorithms. Quadratic discriminant analysis (QDA) was used to examine these six variables to determine which group the record for each dog fell into. QDA generates a quadratic surface in the feature space to separate two or more classes. In a probabilistic setting where four-class QDA corresponds to minimum-error-probability classification of samples from four multivariate Gaussian sub-populations, this parameter reflects the relative prior probabilities of the four classes.

A predictive index was constructed as a simple score system by modelling the posterior probabilities generated from QDA (i.e. p_x_ (%) = posterior probability of being classified into functional ‘Grade X’, p_0_ + p_I_ + p_II_ + p_III_ = 100%) with weights for the relative disease severity added for each posterior probability. The interval of disease severity was assumed to be equal. The score range is from 0 (the centre of the Grade 0 population) to 100% (the centre of the Grade III population):

$$BOAS\;Index\;\left(\%\right)\;=\;0*p_{0}+\;1/3*p_{I}+\;2/3*p_{II}+\;1*p_{III}$$

Binary classification was used to discriminate between BOAS- and BOAS+ FB. The diagnostic value of the BOAS index was assessed by calculating the area under the receiver operating characteristic (ROC) curve. The performance metrics were computed over 2,000 bootstrap samples of the whole dataset to generate the confidence intervals to delineate the expected range of classifier performance and a cross-validation test was applied. Diagnostic accuracy was calculated as sensitivity, specificity, and positive and negative predictive values.

The cut off value selected from the ROC curve was that which best identified significant BOAS where the sensitivity and specificity are approximately equal. The predictive accuracy of the model was then tested in the test group. Functional grades of dogs in the test group were initially withheld to permit blinded application of our algorithms to these records, and then revealed to allow final evaluation of performance. Computations for data processing, feature extraction, QDA, bootstrap resampling, and ROC construction were implemented using the packages “MASS”, “verification”, and “caret” available in the R Project (software platform R 3.1.1. for Mac OS X GUI, http://www.R-project.org).

## Results

### Subjects Characteristics

Eight-nine FB (19/89 ‘patient FB’ and 70/89 ‘study FB’) and 20 non-brachycephalic controls were included in the study. Subject characteristics are given in Table 2. The prevalence of BOAS+ within the total sample group (n = 89) in this study was calculated as 0.54 (95% CI: 0.43–0.65); prevalence of 0.43 (95% CI: 0.31–0.55) was calculated for the study FB (n = 70). 18/30 (60%) of the owners of the BOAS+ study FB reported their dogs never or rarely produced loud respiratory noise and/or breathing difficulty during exercise. There was a significant association between gender and BOAS status, male FB having a prevalence risk ratio of BOAS+ 1.90 times higher than female FB (95% CI = 1.28 to 2.82, p<0.01). BCS showed a small increase with grade in functional tests (mean BCS = 5.0 in Grade 0, 5.29 in Grade I, 5.72 in Grade II and 6.00 in Grade III). BOAS- FB had a significantly lower BCS than BOAS+ FB (mean BCS = 5.23 and 5.79, respectively, p<0.01). Age was not significantly associated with BOAS status.

**Table 2:** Signalment (median [minimum-maximum]) and details of French bulldogs and non-brachycephalic controls.

	French bulldogs	Non-brachycephalic controls ^b^
**Dog number**	89	20
**Female (%)**	58.43	65.0
**Age (years)**	2.5 (1–11)	3.13 (1–12)
**Body weight (kg)**	11.5 (9–17)	13.55 (6.7–27)
**BCS (1–9)**	6 (4–8)	N/A
**Functional grade** ^a^	0 (n = 9, 10.11%); I (n = 32, 35.96%); II (n = 35, 39.33%); III (n = 13, 14.61%);	0 (n = 20)
**Subject type**	Patient dogs (n = 19); Study dogs (n = 70)	Study dogs (n = 20)

Data are presented as median (minimum-maximum). BOAS refers to Brachycephalic Obstructive Airway Syndrome; BCS, body condition score.

^**a**^ Functional grades, refer to Table 1.

^**b**^ Breeds: English springer spaniel (n = 2), Border collie (n = 1), Jack Russell terrier (n = 1), Labrador retriever (n = 3), American bullterrier (n = 1), Beagles (n = 6), Dachshund (n = 1), Cairn terrier (n = 1), West Highland white terrier (n = 1), cross breeds (n = 3).

### Respiratory patterns in FB and non-brachycephalic controls

**Fig 4:**
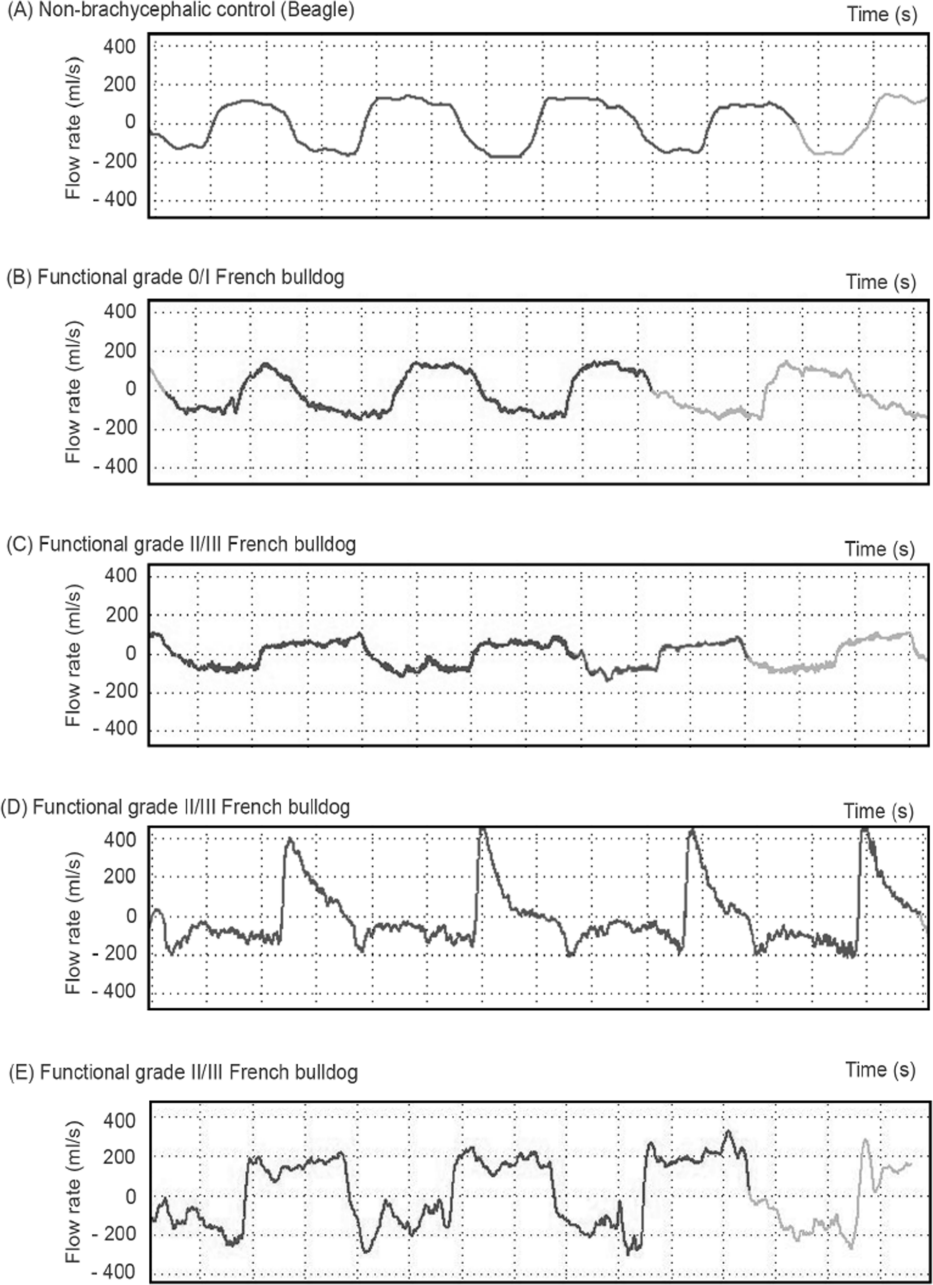
Representative WBBP flow waveforms for several study dogs. (A) non-brachycephalic control dog; (B) BOAS- French bulldog; (C) BOAS+ French bulldog, respiratory cycle Type 1; (D) BOAS+ French bulldog, Type 2; (E) BOAS+ French bulldog, Type 3.

Fig 4 shows the representative constant real-time flow traces with resting RR of non-brachycephalic controls, BOAS- FB and BOAS+ FB. The BOAS+ FB show higher variability in the flow trace characteristics compared to the other two groups, with three main types of flow trace being observed. Type 1 results from restrictive airflow both during inspiration and expiration (flat and square shape, Fig 4C); Type 2 presents with restrictive flow during inspiration but a high peak flow rate during the early expiratory phase followed by an immediate levelling off of flow rate (Fig 4D). Type 3 presents with considerable fluctuating inspiratory airway flow but no significant high peaks during expiration (Fig 4E). Some BOAS+ FB display a mixture of the three types of trace. Noise/unstable flow (i.e. low amplitude, high frequency variation in flow rates) is seen overlying the respiratory cycles of most BOAS+ FB.

**Fig 5:**
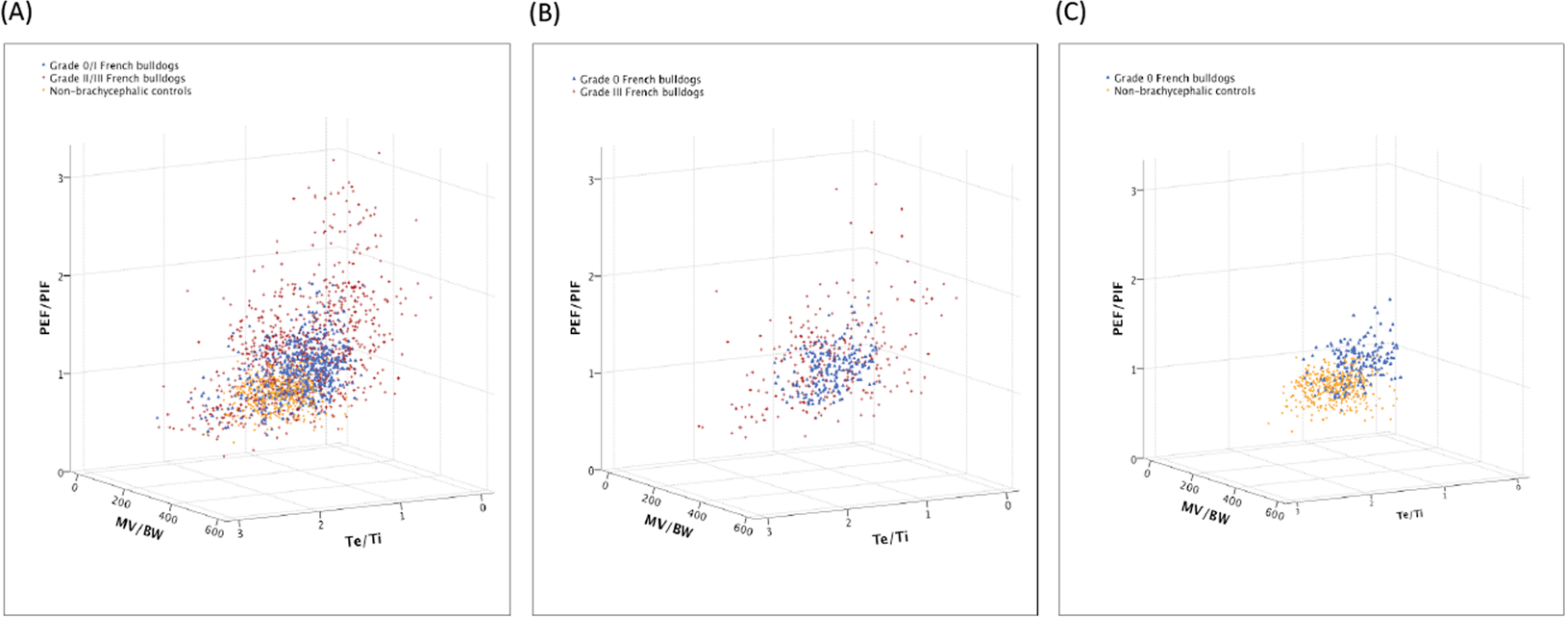
Breaths plotted against three selected respiratory parameters. FB = French bulldogs; PEF/PIF = peak expiratory flow rate/ peak inspiratory flow rate; MV/BW = minute ventilation/ body weight, ml/kg; Te/Ti = expiratory time/ inspiratory time. 20 representative breaths per dog.

Single breath respiratory cycles from all participating dogs were plotted in Fig 5 against the selected features of Te/Ti, PEF/PIF, and MV/BW. The BOAS+ FB show higher variability between dogs and within dogs when compared to BOAS- FB and non-brachycephalic controls (Fig 5A). Breaths plots of the two extreme FB groups (i.e. Grade III and Grade 0 FB) are shown in Fig 5B. Fig 5C compares Grade 0 FB controls and non-brachycephalic controls. The Grade 0 FB controls show a partial overlap in distribution of the breath plots with non-brachycephalic control dogs.

Nonetheless, there are measureable differences in average breathing cycles between these Grade 0 FB controls and non-brachycephalic controls; and between BOAS- FB and BOAS+ FB (Table 3). The Grade 0 FB controls have a significantly lower mean of Te/Ti and higher mean of PEF/PIF than non-brachycephalic controls. A slight trend to reduced MV/BW in the Grade 0 FB controls compared with non-brachycephalic controls was not significant. Variations (i.e. standard deviations) in the selected parameters were not significantly different between the Grade 0 FB controls and non-brachycephalic controls. BOAS+ FB showed on average a 33% elevation in PEF/PIF (p<0.001) and a 12% elevated MV/BW (p<0.05) when compared to BOAS- FB. For both parameters, standard deviations were also elevated significantly. Variation of Te/Ti was significantly higher (p<0.01) in BOAS+ FB but mean Te/Ti was not significantly different from BOAS- FB.

**Table 3:** Respiratory parameters measured by whole-body barometric plethysmography (WBBP).

	Comparison 1		Comparison 2	
	Non-brachycephalic controls^a^ (n = 20)	Grade 0 French bulldog controls (n = 9)	BOAS—French bulldogs^b^ (n = 41)	BOAS + French bulldogs^b^ (n = 48)
**RR_m**	20.85 ± 2.22	22.55 ± 5.87 *	23.13 ± 2.89	22.55 ± 5.04
**RR_sd**	5.48 ± 1.22	2.84 ± 1.46	2.89 ±1.19	3.27 ± 1.38
**TV/BW_m**	8.77±1.98	12.10±3.25**	9.96±2.57	11.05±3.38
**TV/BW_sd**	1.30±0.35	1.66±0.49	1.64±0.62	2.16±0.98 ^τ τ^
**MV/BW_m**	236.83 ± 32.35	213.70 ± 27.78	215.42 ± 24.53	241.46 ± 76.31 ^τ^
**MV/BW_sd**	28.93 ± 8.43	25.07 ± 10.79	26.27 ± 8.88	40.31 ± 19.01 ^τ τ τ^
**Te/Ti_m**	1.36 ± 0.16	0.962 ± 0.18 ***	1.06 ± 0.27 ^π^	1.07 ± 0.39
**Te/Ti_sd**	0.20 ± 0.06	0.174 ± 0.06	0.20 ± 0.07	0.29 ± 0.15 ^τ τ,^ ^π^
**PEF/PIF_m**	0.84 ± 0.08	1.04 ± 0.14 ***	0.99 ± 0.15	1.32 ± 0.42 ^τ τ τ^
**PEF/PIF_sd**	0.12 ± 0.04	0.142 ± 0.04	0.17 ± 0.06	0.32 ± 0.18 ^τ τ τ,^ ^π^

Means (m) and standard deviations of the means (sd) of RR, Te/Ti, PEF/PIF, and MV/BW were calculated for 20 breaths collected from each dog.

RR = respiratory rate (breath per minute); Te/Ti = expiratory time (s) /inspiratory time(s); PEF/PIF = peak expiratory flow rate (ml/s)/ peak inspiratory flow rate (ml/s); MV/BW = minute volume (ml)/ body weight(kg); m = mean of the parameter calculated from the 20 breaths of each dog; sd = standard deviation of the parameter calculated from the 20 breaths of each dog.

^a^ Breeds: English springer spaniel (n = 2), Border collie (n = 1), Jack Russell terrier (n = 1), Labrador retriever (n = 3), American bullterrier (n = 1), Beagles (n = 6), Dachshund (n = 1), Cairn terrier (n = 1), West Highland white terrier (n = 1), cross breeds (n = 3).

^b^ BOAS- FB = Grade 0/I FB; BOAS+ FB = Grade II/III FB. Functional grades, refer to Table 1.

^π^ The Kolmogorov-Smironov test was significant (i.e. data were not normal distributed); p<0.05.

* Significantly different from the non-brachycephalic control group, *p<0.05; **p<0.01; ***p<0.001.

^τ^ Significantly different from the BOAS- French bulldogs; ^τ^ p<0.05; ^τ τ^ p<0.01; ^τ τ τ^ p<0.001.

### QDA Classifier performance and Evaluation of BOAS Index

The FB subjects were divided into 2 datasets: a training dataset of 69 FB, and a test dataset of 20 FB. The QDA results show the classification accuracy for four-group (i.e. Functional Grade 0, I, II, and III) discrimination was 84.06%; for binary discrimination it was 94.2% (i.e. BOAS- versus BOAS+). No Grade 0 FB were misclassified into BOAS+ group; none of the Grade III FB were misclassified into BOAS- (S2 File).

**Fig 6:**
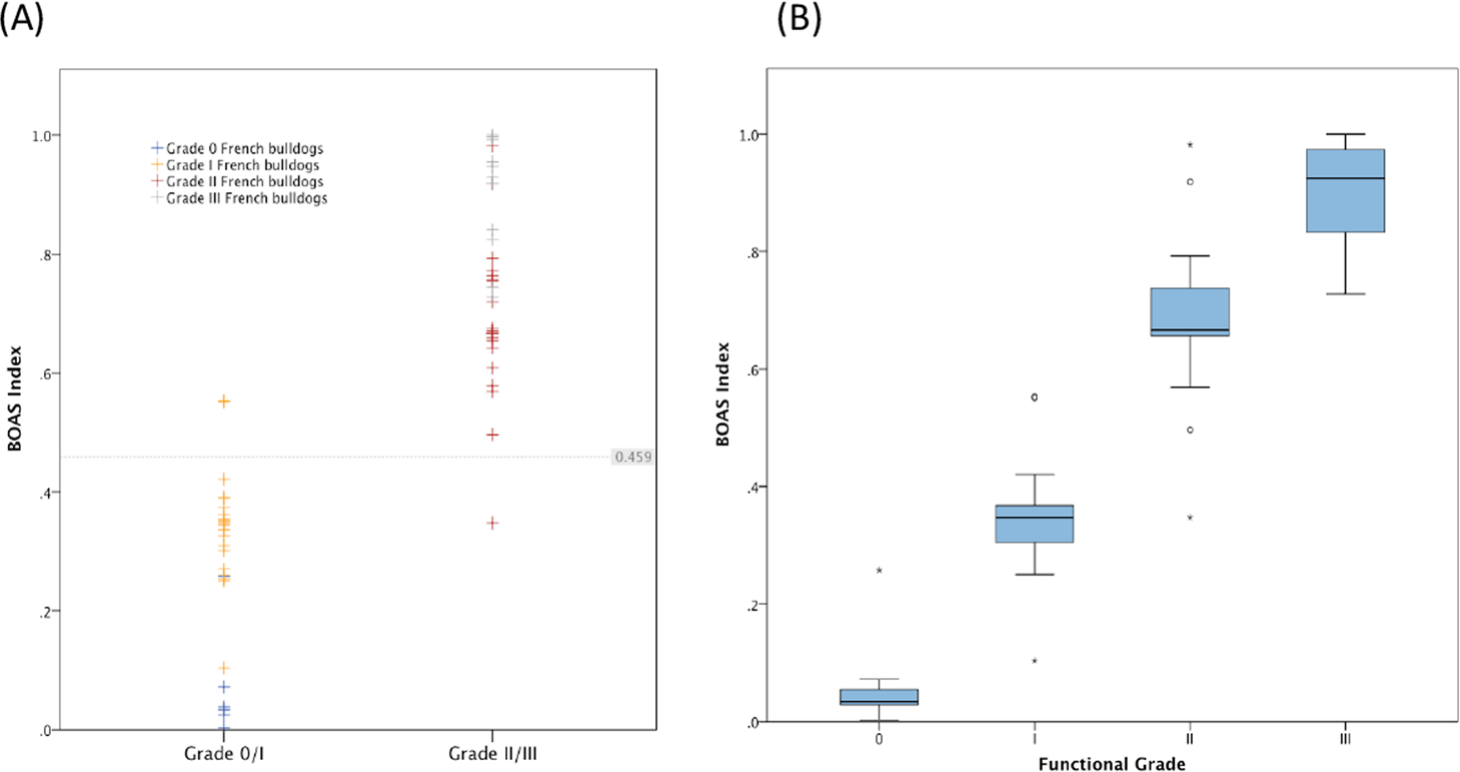
Classification performance of the BOAS Index. (A) Distribution of the BOAS Index for the French bulldog training dataset; (B) Box plots of the BOAS Index for the French bulldog training dataset according to functional grade. Boxes present lines at median, upper and lower quartiles; between whiskers = 95% confidence interval; circles = outliers within the inner fence; stars = outliers within the outer fence.

**Fig 7:**
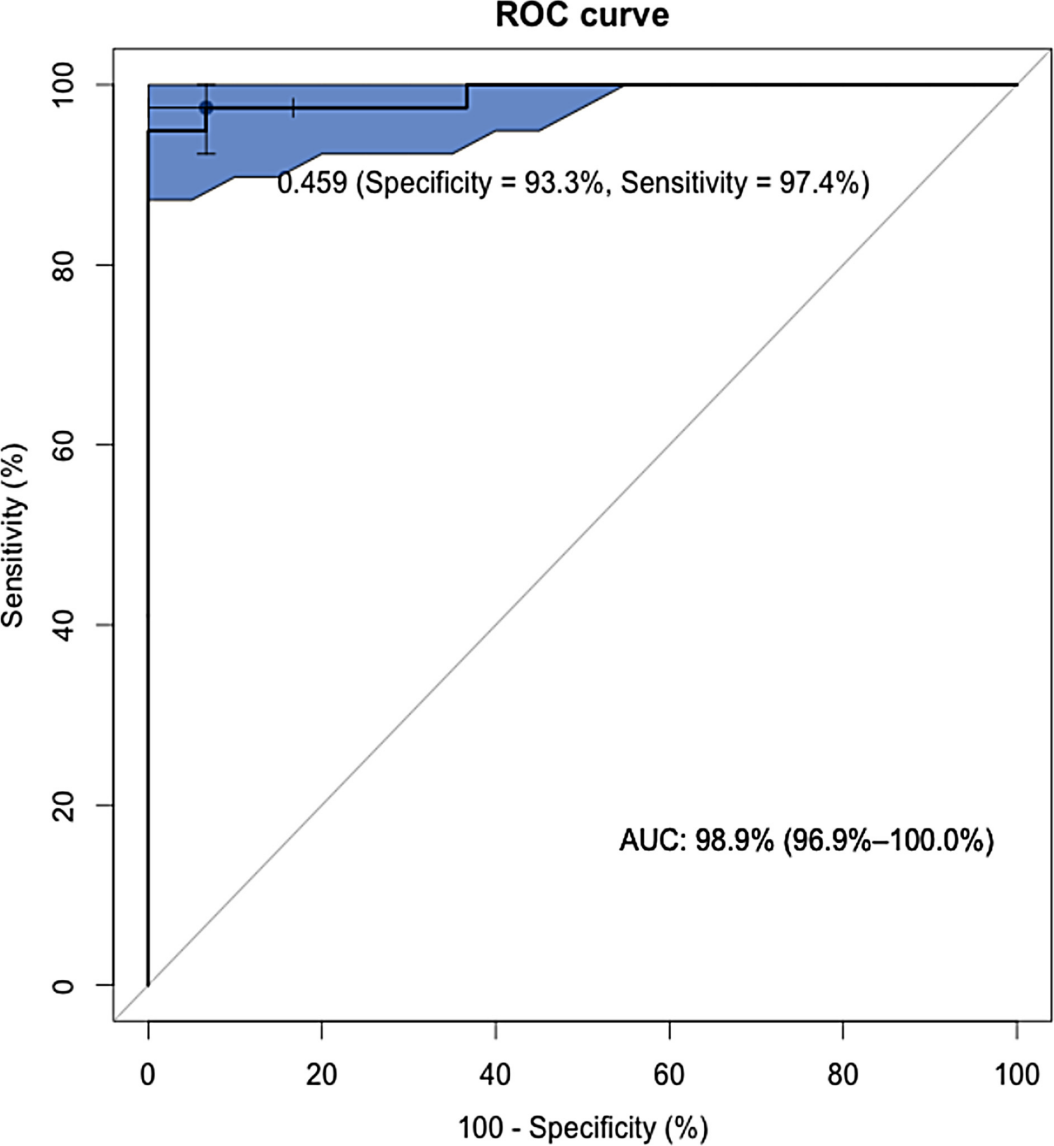
Receiver operating characteristic (ROC) curve of the BOAS Index for diagnosis of functional BOAS+ French bulldogs. Bootstrapping was used to generate the associated 95% confidence intervals (area in blue) to delineate the expected range of screening performance. The black dot with whiskers (95% confidence interval) shows the position of the BOAS Index of 0.46 suggested as a cut off point for distinguishing functionally BOAS- and BOAS+ French bulldogs.

A BOAS Index was calculated for each member of the FB training group (Fig 6). The diagnostic value of this index was assessed in the training group by compiling a ROC curve, showing an area under the curve of 0.99 (95% CI: 0.97–1.0), which indicates a high accuracy of the test (Fig 7). The cut-off value (BOAS index = 0.46) was chosen to yield approximately equal sensitivity (0.97, 95%CI: 0.85–1.0) and specificity (0.93, 95%CI: 0.76–0.99). Classification accuracy was 0.96. Positive predictive value and negative predictive value were 0.95 and 0.97, respectively. When the model was applied to the test dataset, 8 out of 9 BOAS+ FB and all BOAS- FB (n = 11) were correctly identified (classification accuracy = 0.95). Table 4 shows the optimized classification performance for both training and test datasets.

**Table 4:** Optimized classification results using BOAS Index (cut-off point BOAS Index = 0.46) for training and test French bulldogs datasets.

		Screening results			
		Training dataset (n = 69)		Test dataset (n = 20)	
		*BOAS-*	*BOAS+*	*BOAS-*	*BOAS+*
**Functional Grading** ^a^	***0/I (BOAS-)***	28 (TN)	2 (FP)	11 (TN)	0 (FP)
	***II/III (BOAS+)***	1 (FN)	38 (TP)	1 (FN)	8 (TP)
***Classification Accuracy***		0.96 (95%CI: 0.91–0.99)		0.95 (95%CI: 0.85–1.0)	
***Sensitivity***		0.97 (95%CI: 0.85–1.0)		0.89 (95%CI: 0.52–0.98)	
***Specificity***		0.93 (95%CI: 0.76–0.99)		1.0 (95%CI: 0.71–1.0)	
***PPV***		0.95 (95%CI: 0.83–0.99)		1.0 (95%CI: 0.63–1.0)	
***NPV***		0.97 (95%CI: 0.82–0.99)		0.92 (95%CI: 0.61–0.99)	

TN = true positive; FP = false positive; FN = false negative; TP = true positive; PPV = positive predictive value; NPV = negative predictive value; BOAS = brachycephalic obstructive airway syndrome.

^a^ Functional grading for BOAS, see Table 1.

## Discussion

In the present study, a novel approach to quantitative WBBP flow trace analysis was developed. The WBBP respiratory cycles of resting FB with known exercise ability were compared with non-brachycephalic controls as well as with each other. A BOAS Index was proposed based on the classifier for the purpose of disease screening.

The FB is one of the breeds most predisposed to BOAS. However, a proportion of FB who are exposed to the risk of having BOAS (i.e. extreme brachycephalic skull dimensions) do not develop respiratory signs that are clinically concerning. In the present study a large sample of FB subjects were from the pet and show dog population. Only about 10% of FB were found to be Grade 0, with most dogs showing at least some degree of airway restriction. But functionally both Grade 0 FB and Grade I FB, comprising nearly half of FB in the study, were defined as BOAS-. The remaining FB were considered functionally BOAS+. These dogs show exercise intolerance or even life-threatening clinical signs. The low recognition of clinical signs by the pet owners emphasizes the need for an objective screening test. 60% of owners of affected study FB were not able to recognize the BOAS clinical signs, which can cause a delay in treatment and further deterioration of the disease.

The higher prevalence of the condition in males confirms the suggestion in a number of studies of BOAS+ dogs in which males outnumber females (observed ratios of male to female in other studies: 1.7–4.45) [4,30–32]. It has been suggested that obesity may play a role in the severity of BOAS [5], and it is well known to play a role in sleep apnoea in human patients [33]. Here we present direct evidence confirming a role for body condition in BOAS. Although the mean of BCS in BOAS + dogs in this population (5.79) is only slightly over ideal weight, FB with lower BCS are less affected by BOAS. Excessive fat tissue within the upper airway may contribute to abnormal waveform characteristics observed in FB.

Upper airway tract dimensions in brachycephalic dogs are different from those of non-brachycephalic dogs. Significant differences were found between the respiratory patterns of non-brachycephalic controls and Grade 0 FB controls. The most marked differences were in parameters considered to be indicators of upper airway restriction (i.e. lower TV/BW, lower Te/Ti, higher PEF/PIF). However, there were substantial overlaps in respiratory characteristics and the MV/BW was not significantly different between non-brachycephalic controls and Grade 0 FB controls. Similar findings on Te/Ti and PEF/PIF were reported in a study using a facemask and pneumotachograph in bulldogs and Boston Terriers [9]. In brachycephalic dogs, in response to increased upper airway resistance, inspiratory muscles contract for a relatively longer Ti than Te resulting in a decrease in Te/Ti [34,35]. During inspiration, negative transmural pressure narrows the airway and reduces PIF; whilst during expiration positive pressure within the airway reduces narrowing, resulting in an increase in PEF relative to PIF [36]. Airflow and intraluminal pressure changes cause variable flow rates during respiration as a result of the dynamic movement of soft tissues. Therefore, the BOAS- FB were compared with BOAS+ FB, rather than using non-brachycephalic dogs.

To the authors’ knowledge, respiratory traces have not previously been characterised within a large sample of brachycephalic dogs of a single breed. Type 2 WBBP traces (Fig 4D) demonstrated in this study were seen in the majority of BOAS+ FB. Comparison with a previous study of breathing patterns recorded in a smaller group of bulldogs suggest there are some differences between bulldogs and FB results from this study. The TBFVL types reported by Amis and Kurpershoek (1986) are similar to the Type 1 and Type 3 traces in this study (Fig 4C and 4E, respectively). But the most common respiratory trace type in BOAS+ FB, Type 2 (Fig 4D), was not described for bulldogs [9]. This may be because of the different airway tract dimensions in these two breeds. Further study on the association between different lesion sites and WBBP flow characteristics, and breeds variations, is on-going.

The variations in Te/Ti, PEF/PIF and MV/BW increased for BOAS+ FB, which is a reflection of chaotic breathing patterns induced by dynamic airway obstructions. The mean of MV/BW also increased for the BOAS+ FB. But the wider spread of each parameter in BOAS+ traces is the dominant change compared with BOAS—traces, when breaths are plotted (Fig 5A). In previous studies looking at respiratory function in brachycephalic dogs, each respiratory parameter has been assessed independently in dogs with BOAS [9,17]. However, univariate analysis cannot not fully characterise the variation in flow traces. Therefore a multivariate analysis with a quadratic boundary between groups was applied to classify BOAS- FB and BOAS+ FB. Several statistical modelling approaches for classification were tested. The QDA application consistently performed well and required only a modest amount of training data.

BOAS is a disease that does not have an absolute gold standard of diagnosis so that the observation of an expert clinician has been considered the only available standard to define the diagnosis. Subjective functional grading may have intra-observer or inter-observer variations and cause misclassification. In this study, a reference standard was constructed by combining multiple test results. By specifying parameters associated with BOAS+ respiratory signs the composite reference method is standardized and easy to use, but misclassification of subjects is likely to remain. Expert functional grading, used as the starting point here, remains subjective, but the WBBP trace of BOAS- FB and BOAS+ FB proved to be easily distinguishable. In the training dataset, there were 3/69 FB that were misclassified compared with our functional assessment. In order to minimise the effect of individual errors on classification, cross-validation and bootstrap resampling were used. This allowed the final performance to be assessed statistically, thus providing bounds on classification accuracy. The BOAS Index is an indirect scale that is based on the QDA classification results. The concept of the BOAS Index is to give a relative disease severity of each dog. According to the external validation using the test dataset of 20 dogs with functional grade withheld, the performance of the classification is excellent. By using the ROC curve, cut-off points can be chosen for different purposes. For instance, for the purpose of breeding selection, a smaller cut-off value of BOAS Index may be selected in order to include all true positives; whilst for the purpose of making a surgical decision, a higher cut-off value of BOAS Index may be picked up in order to avoid false positives.

There are potential limitations to this study. Firstly, although 3 typical types of waveforms were observed from BOAS+ FB, we have not yet been able to correlate the types of waveforms and lesion sites. One reason is that BOAS is a multi-lesion and progressive disease that may lead to wide variations in respiratory performance. Another difficulty is that under the UK Veterinary Surgeons Act, 1966, and for the welfare benefit of individual FB patients, we are not able to perform either CT scans of the head or endoscopic imaging from the throat and nose from the BOAS- study FB unless the latter had a different relevant clinical condition. Therefore, we were not able to compare the differences in airway dimensions between the group of BOAS- and BOAS+ FB. Secondly, although the effect of external environmental factors on WBBP was minimized by thorough calibration before each recording, variations in factors such as temperature and humidity on the day of testing affect the physical condition of the dog to some extent. Despite these limitations, this preliminary study correctly classified over 96% of FB using WBBP alone. Finally, all of the WBBP data were collected from the wakeful state only. Some of the participating dogs fell asleep at the end of the test, but the data collected during sleeping were not used in this study. Although none of the FB included in this study had any history of sleep apnoea, brachycephalic dogs, particularly bulldogs, have been reported as a naturally-occurring model of obstructive sleep apnoea [37]. Further study on the WBBP traces in BOAS-affected dogs versus non-affected brachycephalic dogs while sleeping is on-going.

## Conclusions

The performance of the QDA classifier for the selected features is highly discriminating, allowing accurate and objective assessment of BOAS status and subsequently an improvement in breed health and clinical decision-making. The classifier in this study is able to provide an objective disease probability for each dog at the time of testing and is able to monitor the disease progress. This study may assist in improving the welfare in FB and other brachycephalic breeds. Due to the non-invasive nature of the technique, it may be used to monitor other respiratory disease and to assess effectiveness of treatment in veterinary medicine and possibly, paediatrics.

## Supporting Information

S1 File Questionnaire used to obtain relevant history from the owners of French bulldogs. (PDF)

S2 File Classification performance for French bulldog training dataset (n = 69) using quadratic discriminant analysis (QDA). (DOCX)
