# Supplementary material for: Characterisation of Brachycephalic Obstructive Airway Syndrome in French Bulldogs Using Whole-Body Barometric Plethysmography
Source: PLoS One. 2015 Jun 16;10(6):e0130741. doi: 10.1371/journal.pone.0130741 (PMC4469695; doi:10.1371/journal.pone.0130741)
Supplement: S1 File — (PDF) [file pone.0130741.s001.pdf]

The Queen's Veterinary School Hospital
University of Cambridge
Madingley Road Cambridge CB3 0ES Tel: 01223 337621

Study no.

BRACHYCEPHALIC STUDY QUESTIONNAIRE

Date: ________________

Owner name: ________________________________

Email address: ____

Contact number:_____________________________________

Address (optional):____

Dog name: _________________________

Breed: ☐ Pug ☐ French bulldog ☐ English bulldog ☐ Others __________________

Kennel club registration number (if applicable): ______________________

Chip number (if applicable): _________________________

Age: _______ year(s) and __________ month(s)

Gender: ☐ Male ☐ Female

Neuter: ☐ Intact ☐ Neutered

Coat colour(s): ______________

Body weight: ____________ Kg

The Queen's Veterinary School Hospital
University of Cambridge
Madingley Road Cambridge CB3 0ES Tel: 01223 337621

Q1. How much exercise does your dog get on average in a day or a week?

________ minute(s)/walk, and ________ walks/day or _______ walks/week

Q2. Does your dog breathe loudly while awake at rest?

☐ Never ☐ Rarely (<once a week) ☐ Frequently (>once a week) ☐ Always

Q2.1. Have you noticed any high pitch noise rather than snorting?

☐ Never ☐ Yes ☐ Not sure

Q3. Does your dog breathe loudly while sleeping?

☐ Never ☐ Rarely (<once a week) ☐ Frequently (>once a week) ☐ Always

Q4. Does your dog breathe loudly during physical exercise?

☐ Never ☐ Rarely (<once a week) ☐ Frequently (>once a week) ☐ Always

Q4.1. Have you noticed any high pitch noise rather than snorting?

☐ Never ☐ Yes ☐ Not sure

Q4.2. Have you noticed inspiratory effort during exercise?

☐ Never ☐ Yes ☐ Not sure

Q5. Has your dog ever had difficulty breathing during exercise or when excited in WINTER?

☐ Never ☐ Rarely (<once a week) ☐ Frequently (>once a week) ☐ Always

Details: ____

Q5.1. How much time does your dog need to recover after physical stress?

☐ up to 5 mins ☐ up to 10 mins ☐ up to 15 mins ☐ up to 20 mins

Q6. Has your dog ever had difficulty breathing during exercise or when excited in SUMMER?

☐ Never ☐ Rarely (<once a week) ☐ Frequently (>once a week) ☐ Always

Details: ____

Q6.1. How much time does your dog need to recover after physical stress?

☐ up to 5 mins ☐ up to 10 mins ☐ up to 15 mins ☐ up to 20 mins

Q7. Has your dog ever collapsed because of dyspnoea?

☐ Never ☐ Yes

Q7.1. If yes, how many times have you noted this episode?

☐ once ☐ twice ☐ more than three times

Q8. Has your dog ever been cyanotic (i.e. blue-purple tongue)?

☐ Never ☐ Rarely (<once a week) ☐ Frequently (>once a week) ☐ Always

Q9. Has your dog ever had reverse sneezing?

☐ Never ☐ Rarely (<once a week) ☐ Frequently (>once a week) ☐ Always

☐ Not sure

Q10. Does your dog have one of the following problems with eating?

Q10.1. Regurgitation/vomiting:

☐ Never ☐ Rarely (<once a week) ☐ Frequently (>once a week) ☐ Always

Q10.2. Choking on food:

☐ Never ☐ Rarely (<once a week) ☐ Frequently (>once a week) ☐ Always

Q10.3. Out of breath during eating:

☐ Never ☐ Rarely (<once a week) ☐ Frequently (>once a week) ☐ Always

Q11. Does your dog have one of the following problems with sleeping?

Q11.1. Wake up a few times during sleep/being almost unable to sleep:

☐ Never ☐ Rarely (<once a week) ☐ Frequently (>once a week) ☐ Always

☐ Not sure

Q11.2. Only able to sleep with the chin in an elevated position:

☐ Never ☐ Rarely (<once a week) ☐ Frequently (>once a week) ☐ Always

☐ Not sure

Q11.3 Attempting to sleep in a sitting position:

☐ Never ☐ Rarely (<once a week) ☐ Frequently (>once a week) ☐ Always

☐ Not sure

The Queen's Veterinary School Hospital
University of Cambridge
Madingley Road Cambridge CB3 0ES Tel: 01223 337621

Q12. Does you dog have any health problem(s) other than respiration?

☐ No ☐ Yes

If yes, details:

____

____

Is your dog on any medication?

____

Q13. Has your dog undergone any surgical procedure(s)?

☐ No ☐ Yes; surgical procedures: __________________________

If yes, when did he/she undergo the surgery?

________ year(s) and __________ month(s) old

The outcome of the surgery:

☐ Very satisfied ☐ Satisfied ☐ Neutral ☐ Dissatisfied ☐ Very dissatisfied

Details:

____

____

The Queen's Veterinary School Hospital
University of Cambridge
Madingley Road Cambridge CB3 0ES Tel: 01223 337621

Please provide any additional comments regarding your dog or this clinical survey

Would you like to receive updates on our study? ☐ No ☐ Yes

Thank you very much for your help!
We greatly appreciate your time and cooperation in filling out this questionnaire.

Nai-Chieh Liu *DVM MPhil*
Clinical PhD candidate
Email address: ncl25@cam.ac.uk

Jane Ladlow *MA VetMB CertVR CertSAS DipECVS MRCVS*
European Specialist in Small Animal Surgery
Email address: jfl1001@cam.ac.uk

David Sargan *MA PhD*
Geneticist
Email address: drs20@cam.ac.uk
