# Supplementary material for: Characterisation of Brachycephalic Obstructive Airway Syndrome in French Bulldogs Using Whole-Body Barometric Plethysmography
Source: PLoS One. 2015 Jun 16;10(6):e0130741. doi: 10.1371/journal.pone.0130741 (PMC4469695; doi:10.1371/journal.pone.0130741)
Supplement: S2 File — (DOCX) [file pone.0130741.s002.docx]

**Classification performance for French bulldog training dataset (n=69) using quadratic discriminant analysis (QDA)**

|  | | | **QDA Classification Output** | | | | |
| --- | --- | --- | --- | --- | --- | --- | --- |
|  |  |  | ***BOAS-*** | | ***BOAS+*** | |  |
|  |  |  | ***0*** | ***I*** | ***II*** | ***III*** | **Total** |
| **Functional Grading^a^** | ***BOAS-*** | ***0*** | 7 | 1 | 0 | 0 | **8** |
|  |  | ***I*** | 1 | 19 | 2 | 0 | **22** |
|  | ***BOAS+*** | ***II*** | 0 | 2 | 23 | 2 | **27** |
|  |  | ***III*** | 0 | 0 | 3 | 9 | **12** |
|  |  | **Total** | **8** | **22** | **28** | **11** | **69** |
| BOAS=brachycephalic obstructive airway syndrome  ^a^ Functional grading for BOAS, see Table 1 | | | | | | | |
